# Supplementary material for: Are climate change perceptions related with plastic policy support? Effects of climate change skepticism, guilt, and efficacy on the acceptance of the plastic tax
Source: PLoS One. 2025 Dec 29;20(12):e0337327. doi: 10.1371/journal.pone.0337327 (PMC12747356; doi:10.1371/journal.pone.0337327)
Supplement: S1 Appendix — (PDF) [file pone.0337327.s001.pdf]

S1. Appendix

Table 1. Moderator analysis (climate change skepticism)

Variables		Model		
		b	S.E	Beta
(Constants)		3.530***	.179	
Sociodemographic factors	Gender	-.018	.041	-.009
	Age	.057***	.015	.087
	Education	.097*	.042	.051
	Income	.029	.024	.026
Independent 1 Values factors	Perceived risk	.218***	.037	.176
	Knowledge	.158***	.033	.129
	Trust	.195***	.036	.148
	Negative emotion	.064	.034	.052
Independent 2 Risk Perception Paradigm	Environmentalism	.153***	.034	.116
	Altruism	.080**	.023	.085
	Egalitarianism	.102***	.023	.096
	Materialism	-.100***	.026	-.093
Moderator variable	Climate change skepticism	-.116***	.027	-.115
Independent × Moderator	Perceived risk*Climate change skepticism	-.048	.037	-.043
	Knowledge*Climate change skepticism	.002	.029	.002

	Trust*Climate change skepticism	-.014	.032	-.013
	Emotion*Climate change skepticism	.082*	.032	.079
	Environmentalism*Climate change skepticism	-.002	.035	-.002
	Altruism*Climate change skepticism	.011	.025	.010
	Egalitarianism*Climate change skepticism	.007	.024	.007
	Materialism*Climate change skepticism	.097**	.024	.096
F value		39.629		
R		0.6		
R^2		0.361		
adj. R^2		0.351		
$^{*}p<.05$, $^{**}p<.01$, $^{***}p<.001$				

Table 2. Moderator Analysis (Guilt)

Variables		Model		
		b	S.E	Beta
(Constants)		3.504***	.179	
Sociodemographic factors	Gender	-.021	.040	-.011
	Age	.059***	.015	.090
	Education	.116**	.042	.062
	Income	.032	.024	.029
Independent 1 Values factors	Perceived risk	.238***	.036	.192
	Knowledge	.094**	.032	.077
	Trust	.143***	.035	.108
	Emotion	.056	.033	.045
Independent 2 Risk Perception Paradigm	Environmentalism	.174***	.035	.131
	Altruism	.100***	.023	.106
	Egalitarianism	.086***	.023	.081
	Materialism	-.096***	.025	-.089
Moderator variable	Guilt	.110**	.033	.089
Independent × Moderator	Perceived risk*Guilt	-.107*	.043	-.088
	Knowledge*Guilt	.037	.034	.031
	Trust*Guilt	-.045	.033	-.040
	Emotion*Guilt	.039	.036	.033
	Environmentalism*Guilt	.075*	.038	.053

	Altruism*Guilt	.057*	.029	.050
	Egalitarianism*Guilt	-.063*	.028	-.050
	Materialism*Guilt	.033	.029	.027
F value		38.937***		
R		0.597		
R^2		0.356		
adj.R^2		0.347		
*p<.05, **p<.01, ***p<.001				

Table 3. Moderator Analysis (Efficacy)

Variables		Model		
		b	S.E	Beta
(Constants)		3.530***	.179	
Sociodemographic factors	Gender	-.015	.040	-.008
	Age	.057***	.015	.087
	Education	.099*	.042	.053
	Income	.031	.024	.028
Independent 1 Values factors	Perceived risk	.240***	.037	.193
	Knowledge	.103**	.032	.085
	Trust	.130***	.036	.099
	Emotion	.058	.033	.047
Independent 2 Risk Perception Paradigm	Environmentalism	.167***	.034	.126
	Altruism	.102***	.023	.108
	Egalitarianism	.086***	.023	.081
	Materialism	-.099***	.025	-.092
Moderator variable	Efficacy	.115**	.034	.095
Independent × Moderator	Perceived risk*Efficacy	-.099*	.043	-.083
	Knowledge*Efficacy	.003	.033	.003
	Trust*Efficacy	-.012	.034	-.011
	Emotion*Efficacy	.044	.036	.038
	Environmentalism*Efficacy	.056	.038	.040

	Altruism*Efficacy	.075**	.029	.065
	Egalitarianism*Efficacy	-.020	.027	-.016
	Materialism*Efficacy	.044	.029	.038
F value		38.531***		
R		0.595		
R^2		0.354		
adj. R^2		0.345		
*p<.05, **p<.01, ***p<.001				
